# Comprehensive Evaluation of Language, Speech, and Communication Skills in 24‐ to 36‐Month‐Old Twins and Singletons: Developmental Differences and Associated Factors

**DOI:** 10.1002/brb3.71669

**Published:** 2026-08-12

**Authors:** Mümüne Merve Parlak, Emrah Karacaoğlu, Cansu Yıldırım Tutar, Oğuz Kadir Eğilmez

**Affiliations:** ^1^ Department of Speech and Language Therapy Faculty of Health Sciences Ankara Yıldırım Beyazıt University Ankara Türkiye; ^2^ Department of Otolaryngology Sakarya University Training and Research Hospital Sakarya Türkiye; ^3^ Department of Speech and Language Therapy Faculty of Health Sciences İzmir Bakırçay University İzmir Türkiye

**Keywords:** children, communication, language development, singleton, speech, twin, twin effect

## Abstract

**Objective:**

Early language development forms the foundation for cognitive, social, and academic skills. This study aimed to compare the language, speech, and communication skills of twins and singleton children aged 24–36 months and to explore associations with sociodemographic, biological, and environmental factors in the Turkish population

**Method:**

The study was conducted with 100 children, comprising 30 twin pairs (60 children, 30 male, 30 female) and 40 singletons (23 male, 17 female), with a mean age of 33.4 ± 5.26 months. Receptive and expressive language skills were evaluated using the Test of Early Language Development—Third Edition, Turkish Version (TEDIL), parent‐reported language skills were assessed with the Adapting the Language Development Survey to Turkish (DILTAR), communication skills were evaluated with the Children's Communication Checklist‐2 (CCC‐2), and speech development was assessed with the Ankara Articulation Test (AAT). Sociodemographic (maternal age, education), biological (birth weight, gestational age), and environmental (television viewing time) factors were analyzed through correlational analyses.

**Results:**

Twins exhibited significantly lower birth weights and earlier gestational age at birth compared to singletons. Twins demonstrated lower performance in TEDIL receptive language scores and AAT scores compared to singletons (*p* < 0.05). In the CCC‐2 subscales of speech, syntax, semantics, use of context, and social relationships, twins scored significantly lower than singletons. A negative correlation was found between TEDIL receptive language scores and television viewing time across all children (*r* = –0.603). In twins, statistically significant correlations were observed between language‐related scores and maternal age, maternal gestational age, birth weight, and maternal education (*p* < 0.05).

**Conclusion:**

This study provided culturally grounded evidence from Turkish children and demonstrated that 24–36‐month‐old twins are at increased risk for delays in language, speech, and communication skills compared to singleton peers. The findings suggest that the twinness effect in early toddlerhood reflects the combined influence of biological maturity at birth (e.g., lower birth weight and earlier gestational age), sociodemographic characteristics (e.g., maternal education and age), and environmental factors (e.g., television viewing time). Rather than being attributable to a single determinant, early language and communication outcomes in twins appear to emerge from dynamic interactions between biological and environmental conditions. These results underscore the importance of early, multidimensional screening and comprehensive assessment of twins’ communication development to facilitate the timely identification of potential delays. Early intervention approaches that combine speech‐language therapy with enriched environmental support strategies may help mitigate developmental risk and promote optimal communication outcomes.

## Graphical abstract

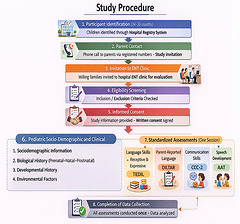

## 1 Introduction

In recent years, there has been a notable increase in twin birth rates worldwide. For instance, in the United States, the twin birth rate rose from 1 in 53 infants (1.8%) in 1980 to 1 in 30 infants (3.3%) in 2009 (Martin et al. 2012). In Türkiye, in 2024, the number of multiple births was 31,109, and 3.3% of births were multiple births, 97.0% of which were twins (TÜİK 2024). Twins face higher risks of mortality and morbidity during the prenatal, perinatal, and neonatal periods, making their developmental outcomes a significant focus for researchers, clinicians, educators, and parents (Blickstein and Keith 2005).

Twins have long been a key subject in studies examining the roles of genetic and environmental factors in language and cognitive development. Twin studies are among the most commonly used methods to assess the interaction between genetics and environment. However, the “twinness effect” on language acquisition remains relatively understudied (Taylor et al. 2018; Thorpe 2006). The “twinness effect” refers to the phenomenon where twins experience delays in language acquisition compared to singletons (Hay et al. 1987; Rutter et al. 2003; Taylor et al. 2018). Dʼhaeseleer et al. (2016) found that twins score lower in expressive and receptive language skills, and a study of 2‐ to 6‐year‐old twins identified delays in language acquisition compared to singleton peers (Dʼhaeseleer et al. 2016). However, some studies suggest that the twinness effect is limited to language acquisition and does not significantly impact speech or nonverbal cognitive skills (Rutter et al. 2003; Thorpe et al. 2003). This variability highlights the need for a more comprehensive and methodologically multifaceted investigation of the developmental outcomes of twins.

Despite growing interest in the twinness effect, important methodological limitations persist in the literature. There are no detailed studies, primarily in the national literature, on speech and language assessments, vocabulary, and grammar skills in twins during infancy and/or early childhood that include age‐specific references. To better understand the twinness effect, the use of norm‐referenced and standardized assessment methods is emphasized (Rice 2020). Some studies have used non‐standardized measures, and even when standardized measures were employed, comparisons with age norms were not the primary focus (Rice et al. 2014). The Twins’ Early Development Study (Oliver and Plomin 2007), one of the population‐based studies on twins’ language development, focused on percentile rankings within twin samples rather than general population norms (Dale et al. 2018; Hayiou‐Thomas et al. 2014). Additionally, prior research frequently encompasses broad age ranges and relatively small sample sizes (Dʼhaeseleer et al. 2016; Kokkinaki et al. 2023), limiting the precision with which early developmental differences can be characterized. Together, these issues restrict a clear understanding of the magnitude and nature of the twinness effect.

Another critical consideration involves identifying the biological, environmental, and sociodemographic factors that may influence speech and language‐related development in twins (Matsumoto et al. 2025; Sunderajan and Kanhere 2019; Viding et al. 2004). Understanding the factors associated with language, speech, and communication skills affected by the twinness effect is essential for planning early intervention programs (Hayashi and Hayakawa 2004; Sapiets et al. 2021). Some studies suggest that preterm birth alone is not the primary cause of language delays, with environmental and social factors also playing significant roles (Dʼhaeseleer et al. 2016; Toseeb et al. 2022). However, no consistent relationship has been found between sociodemographic risk factors (e.g., low maternal education, socioeconomic disadvantage) and delayed language development in twins (Rice et al. 2014; Taylor et al. 2018). Furthermore, the lack of studies examining factors potentially related to language, speech, and communication skills in Turkish‐speaking twin populations constitutes a significant gap in the literature.

Early speech, language, and communication development provides the foundation for later cognitive, social, and academic functioning (Akagündüz et al. 2023; Çelik et al. 2025). However, most prior studies focused solely on language skills and did not simultaneously evaluate speech and pragmatic communication abilities. Addressing this limitation is essential for achieving a holistic understanding of early speech, language and communication development in twins. Moreover, given the significant role of cultural factors in language development (Hoff and Tian 2005; Marshall 2000), the lack of comprehensive studies in the Turkish population investigating the twinness effect on language, speech, and communication skills is noteworthy.

Focusing on the 24‐ to 36‐month period is particularly important, as this stage falls within an early developmental window characterized by substantial neuroplasticity and rapid expansion in vocabulary, grammar, and emerging pragmatic skills (Kuhl 2010; Pine 2005; Tomasello 2003), making it a sensitive period for detecting early variations in language, speech, and communication development (Fenson et al. 1994). Identifying delays during this period is essential, as early intervention initiated in the first years of life is associated with the most favorable long‐term outcomes (Guralnick 2011; Roberts and Kaiser 2011). To the best of our knowledge, no previous study has simultaneously assessed language, speech, and communication skills in twins and singletons within a specific early age range using standardized, norm‐referenced instruments, while also examining the sociodemographic, biological, and environmental factors potentially associated with developmental outcomes. Given that early communication patterns are closely shaped by cultural context, the absence of comprehensive research conducted in Turkish‐speaking populations constitutes a notable gap in the literature.

Investigating these developmental domains in twins within the Turkish context carries particular relevance, given both the scarcity of relevant studies in this population and the relatively high frequency of twin births among multiple pregnancies reported in Türkiye (TÜİK 2024), which renders this group both clinically and culturally significant. Addressing this gap is therefore critical, not only for achieving a holistic understanding of early speech, language, and communication development in twins, but also for accurately characterizing the developmental profiles associated with twinship and informing the design of culturally appropriate early intervention strategies. Therefore, the present study aimed to comprehensively assess the language, speech, and communication skills of 24‐ to 36‐month‐old twin children, compare their performance with singleton peers, and identify the sociodemographic, biological, and environmental factors associated with developmental outcomes.

In line with these aims, the study addressed the following research questions:

1. What are the language, speech, and communication skill profiles of twin children aged 24–36 months based on standardized, norm‐referenced assessments?
2. Do twin children differ significantly from singleton peers in language, speech, and communication performance?
3. Are receptive and expressive language skills, speech production, and pragmatic communication abilities differentially affected within the twin group?
4. Which sociodemographic (e.g., maternal education), biological (e.g., birth weight, gestational age), and environmental (e.g., screen exposure) factors are associated with performance in language, speech, and communication domains?

## 2 Method

This study was approved by the Non‐Invasive Clinical Research Ethics Committee of Sakarya University Faculty of Medicine (Ethics Committee Date: 21.11.2024, No: 173). The study was conducted at the Department of Ear, Nose and Throat (ENT), Head and Neck Surgery at the hospital.

### 2.1 Study Procedure

**FIGURE 1:**
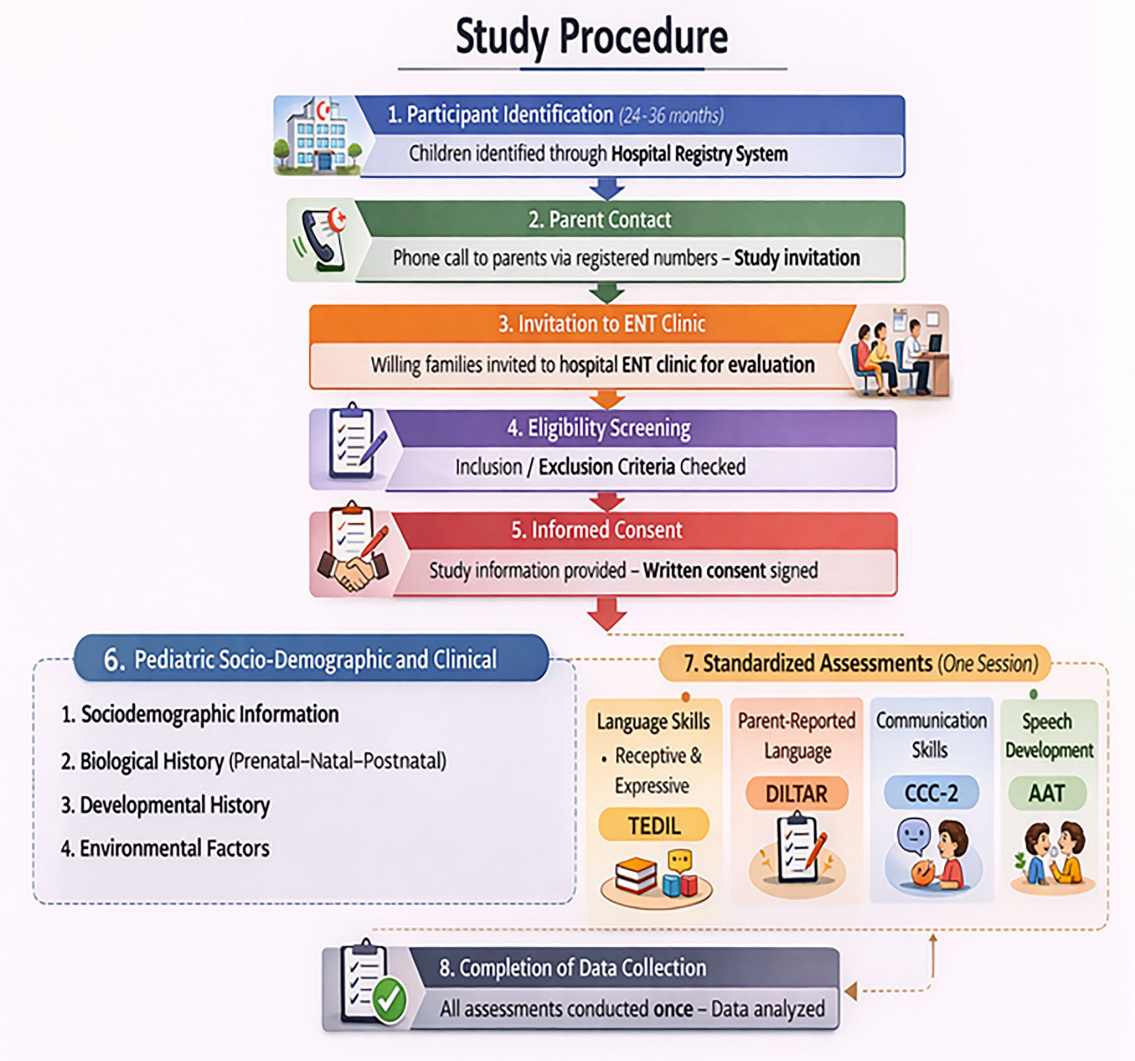
Study procedure.

Children aged 24–36 months were identified through the hospital's registry system. Parents were contacted via the phone numbers provided in the system and invited to participate. Willing parents and their children were invited to the ENT clinic for evaluation. All participants who met the inclusion criteria and consented to continue were informed about the study, and parents were asked to sign informed consent forms. The data collection process began with the administration of the Pediatric Socio‐Demographic and Clinical Information Form, through a family interview conducted with the parents. Children meeting the inclusion criteria (see below) underwent detailed assessments of language, speech, and communication skills. Receptive and expressive language skills were evaluated using the Test of Early Language Development—Third Edition, Turkish Version (TEDIL), parent‐reported language skills were assessed with the Adapting the Language Development Survey to Turkish (DILTAR), communication skills were evaluated with the Children's Communication Checklist‐2 (CCC‐2), and speech development was assessed with the Ankara Articulation Test (AAT). Assessments were conducted once. The study procedure is summarized in Figure 1.

### 2.2 Participants

Inclusion criteria included normal bilateral peripheral hearing, chronological age between 24 and 36 months, Turkish as the primary language, no additional disabilities, and no prior language or speech therapy. Exclusion criteria were the presence of additional diagnosed disabilities, a primary language other than Turkish, and hearing loss.

**FIGURE 2:**
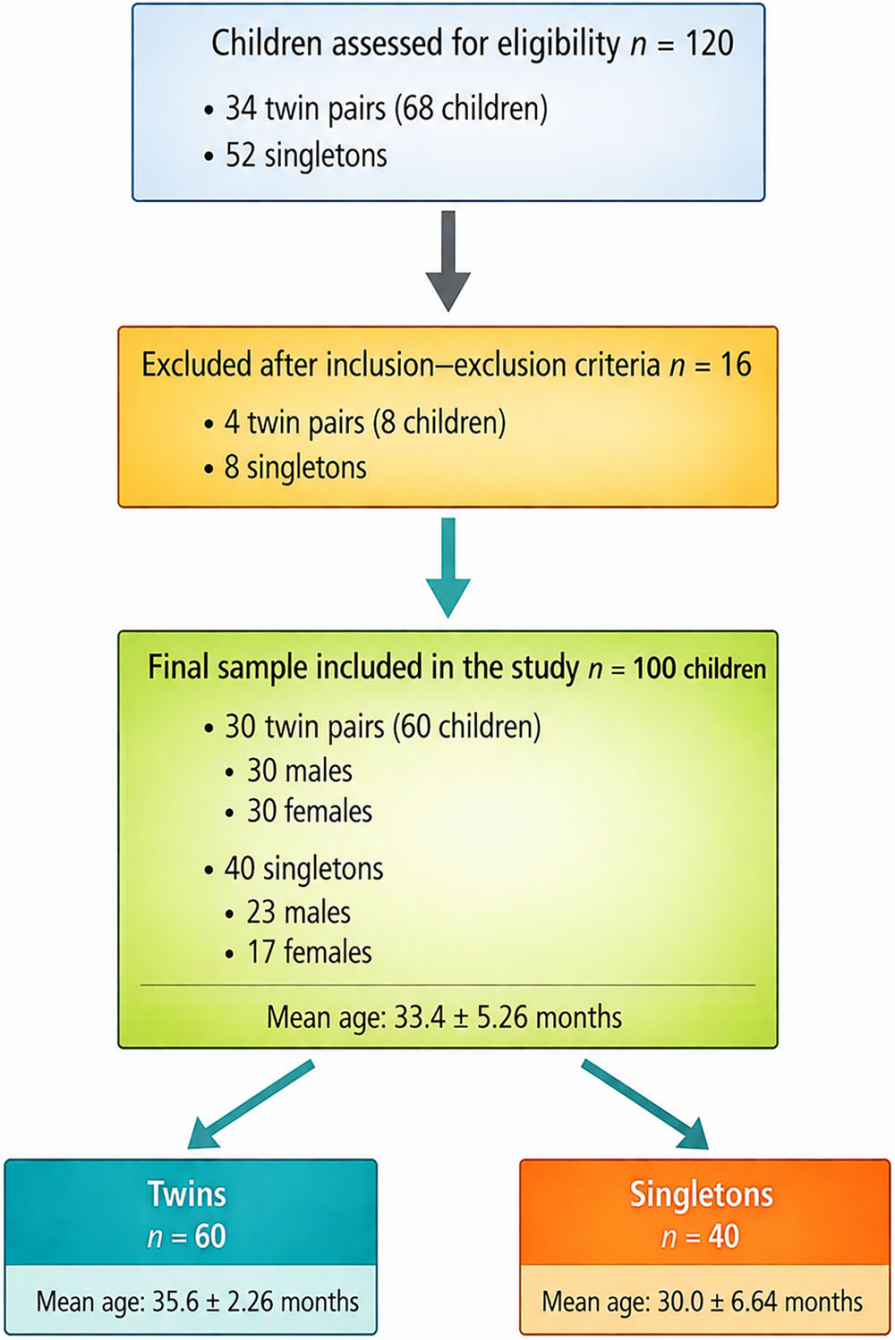
Participant selection and final sample characteristics of twin and singleton children.

The study initially evaluated 120 children, including 34 twin pairs (68 children) and 52 singletons. After applying the inclusion and exclusion criteria, the study was completed with 100 children: 30 twin pairs (60 children, 30 males, 30 females) and 40 singletons (23 males, 17 females), with a mean age of 33.4 ± 5.26 months. Twins had a mean age of 35.6 ± 2.26 months, while singletons had a mean age of 30.0 ± 6.64 months (see Figure 2). The classification of the twins according to zygosity was conducted based on the information provided to the families following prenatal ultrasonographic evaluations and parental report; 18 (60%) of the sample were monozygotic and 12 (40%) were dizygotic twins.

### 2.3 Data Collection Tools

#### 2.3.1 Pediatric Socio‐Demographic and Clinical Information Form

The Pediatric Socio‐Demographic and Clinical Information Form was designed to comprehensively assess children's medical history, developmental characteristics, language and speech skills, and environmental risk factors. Routinely used in clinical practice, the form was administered in a standardized manner within the scope of the study, and additional variables deemed necessary for the research objectives were incorporated. Based on previous literature indicating that sociodemographic factors (e.g., maternal education level), environmental factors (e.g., television viewing time), biological factors (e.g., birth weight), and genetic factors may be significantly associated with developmental delays, these variables were categorized separately in the study. Accordingly, the form consisted of four main sections.

The first section included sociodemographic information about the family. The age, educational level, occupation, city of residence, and primary caregiver information of both the mother and father were recorded. Maternal and paternal education levels and parental occupations were analyzed as sociodemographic variables.

The second section collected prenatal, natal, and postnatal medical information, including gestational age, mode of delivery, birth weight, history of anoxia, hyperbilirubinemia, hearing screening results, and other relevant medical conditions. Birth weight and gestational age were evaluated as biological factors.

The third section addressed developmental history in detail, including motor and language developmental milestones, age of first word production, current communication level, and speech intelligibility. Parents were also asked whether there was a history of developmental delay among parents or siblings in the family. Family history of developmental delay and zygosity status were evaluated as genetic factors. In line with the characteristics of the sample, a zygosity section was added to the form. Zygosity was recorded as monozygotic or dizygotic based on information provided to the family following prenatal ultrasonographic evaluation and parental report.

The fourth section focused on environmental variables. Television viewing status (present/absent) and average daily television viewing time (minutes/hours) were recorded, along with tablet/phone use, time spent playing with the child, and frequency of shared book reading. Daily television viewing time and overall screen exposure were analyzed as environmental factors.

The form was administered through face‐to‐face interviews with parents, and responses were recorded by the researcher. Administration took approximately 20–30 min. During data analysis, variables were classified as biological (birth weight, gestational age), genetic (family history of developmental delay, zygosity), sociodemographic (maternal and paternal education level, occupation), and environmental (television viewing time, screen exposure, and time spent in home‐based interaction).

#### 2.3.2 Test of Early Language Development—Third Edition, Turkish Version (TEDIL)

The Turkish adaptation of the Test of Early Language Development–Third Edition (“Hresko et al. (1999). Test of Early Language Development (TELD) Third Edition. PRO‐ED. Austin:Texas”), known as TEDIL, was developed by Güven and Topbaş (2014). TEDIL assesses language development in children aged 2;0–7;11 years and includes subtests for receptive and expressive language skills. It evaluates semantic, morphological, and syntactic development compared to age norms, using raw scores, standard scores, and equivalent ages (Güven and Topbaş 2014). Raw scores from the Receptive and Expressive Language subtests are calculated based on correct responses and converted to standard scores and equivalent ages using the test manual.

#### 2.3.3 Adapting the Language Development Survey to Turkish (DILTAR)

The Language Development Survey, developed by Rescorla (1989), was adapted into Turkish as DILTAR by Topbaş et al. (2016), and its validity and reliability were established. DILTAR is an effective tool for identifying delayed language and speech issues, with 90% sensitivity and 97.9% specificity. It includes a brief developmental information form and a list of 313 words across 14 semantic categories (e.g., food, animals, vehicles)(Topbaş et al. 2016). Parents are asked to mark words their child spontaneously produces. DILTAR is a robust tool for predicting language skills in 24‐ to 35‐month‐old children with delayed language development (Topbaş et al. 2016).

#### 2.3.4 Children's Communication Checklist‐2 (CCC‐2)

Bishop (2013) confirmed the validity and reliability of the CCC‐2 for assessing communication disorders, including language and speech. CCC‐2 was adapted into Turkish by Ünal and Şahin (2022). The CCC‐2 screens communication skills in children with language limitations, identifying pragmatic language issues. It is completed by parents, caregivers, or educators who have been in regular contact with the child for at least three months. The scale, which takes approximately 10–15 min to complete, consists of 70 items across 10 subscales: speech, syntax, semantics, coherence, appropriateness of speech, stereotyped language, use of context, nonverbal communication, social relations, and interests (Ünal and Şahin 2022).

#### 2.3.5 Ankara Articulation Test (AAT)

Developed by Ege et al. (2005), the AAT assesses articulation skills in 2‐ to 12‐year‐old children for screening and therapeutic purposes. The test has content and structural validity and requires children to name age‐appropriate colored pictures. Each sound is evaluated in five positions: word‐initial (before a vowel), word‐final (after a vowel), syllable‐initial (after a consonant), syllable‐final (before a consonant), and between two vowels. Productions are recorded, errors are calculated, and raw scores are converted to standard scores based on age‐specific tables (Ege et al. 2005).

### 2.4 Data Analysis

The data were analyzed using SPSS 22 and Jamovi 2.3.28 software. Descriptive statistics for categorical variables were reported as counts and percentages, while normally distributed numerical variables were presented as means and standard deviations, and non‐normally distributed numerical variables as medians. AAT, CCC‐2, TEDIL, and DILTAR results and potentially related factors were compared between twin and singleton groups using Independent samples *t*‐test or Mann–Whitney *U* test, depending on the normality of the data distribution. The associations between environmental, sociodemographic, and biological factors and the numerical variables of AAT, CCC‐2, TEDIL, and DILTAR results were analyzed using Pearson or Spearman correlation tests based on distribution normality. Correlation coefficients of ±0.70–1.00 indicated high correlation, ±0.30–0.70 moderate correlation, and ±0.00–0.30 low correlation (Gürbüz and Şahin 2014). The significance level was set at 0.05.

## 3 Results

The mean birth weight of twins was 2693 ± 631 grams, with a mean gestational age of 36.2 ± 2.40 weeks. Singletons had a mean birth weight of 3295 ± 524 grams and a mean gestational age of 38.5 ± 1.86 weeks. Statistically significant differences were observed between twins and singletons in terms of age, birth weight, and gestational age (*p* < 0.001). The average daily television viewing time among twins was 181 ± 121 min, which was significantly higher than that of singletons (*p* = 0.015) (Table 1).

**TABLE 1:** Comparison of factors potentially affecting language, speech, and communication skills in twin and singleton children.

	Twins Mean ± SD (median)	Singletons Mean ± SD (median)	*p* value
Child's age (months)	35.6 ± 2.26 (36.0)	30.0 ± 6.64 (30.0)	**<0.001**
Child's birth weight (grams)	2693 ± 631 (2780)	3295 ± 524 (3290)	**<0.001^a^**
Child's gestational week at birth	36.2 ± 2.40 (37.0)	38.5 ± 1.86 (39.0)	**<0.001**
Maternal gestational age	29.5 ± 6.12 (27.5)	29.2 ± 3.36 (29)	0.399
Maternal age	32.9 ± 6.62 (30.5)	31.8 ± 3.42 (32.0)	0.213
Maternal education level (years)	11.3 ± 3.91 (12.0)	11.4 ± 5.32 (12)	0.442
Television watching (minutes/day)	181 ± 121.0 (120)	117 ± 98.1 (100)	**0.015**

^a^Independent samples *t*‐test, the bold values are considered statistically significant.

Twins had a TEDIL receptive language standard score of 102.0 ± 15.0, an expressive language score of 103.0 ± 18.0, a DILTAR mean word count of 131.0 ± 123.0, and an AAT standard score of 86.5 ± 13.2. Singletons scored significantly higher than twins in TEDIL receptive language standard scores (*p* = 0.007) and in AAT standard scores (p = 0.001), while twins showed significantly higher AAT raw scores (i.e., more articulation errors) than singletons (p = 0.037). In the CCC‐2 subscales of speech, syntax, semantics, use of context, and social relationships, twins scored significantly lower than singletons (*p* < 0.05) (Table 2).

**TABLE 2:** Comparison of TEDIL, AAT, and CCC‐2 results between twin and singleton children.

	Twins mean ± SD (median)	Singletons mean ± SD (median)	*p* value
TEDIL receptive language raw scores	15.5 ± 3.65 (17)	17.8 ± 4.49 (19)	0.055
TEDIL receptive language standard scores	102.0 ± 15.0 (105.5)	118.0 ± 19.4 (120)	**0.007**
TEDIL expressive language raw scores	17.7 ± 4.55 (18)	19.1 ± 6.26 (21.5)	0.218
TEDIL expressive language standard scores	103.0 ± 18.0 (99)	106.0 ± 37.1 (116)	0.369**^a^**
DILTAR	131.0 ± 123.0 (101)	139 ± 106 (155)	0.054
AAT raw score	39.1 ± 21.4 (32.5)	25.7 ± 14.5 (22.5)	**0.037**
AAT standard score	86.5 ± 13.2 (88.5)	103 ± 12.0 (105.5)	**0.001**
CCC2—Speech	6.00 ± 3.07 (6)	8.15 ± 3.77 (8)	**0.003^a^**
CCC2—syntax	3.10 ± 2.05 (3)	4.33 ± 2.64 (5)	**0.010**
CCC2—semantics	3.57 ± 3.20 (3)	5.15 ± 3.08 (5)	**0.014**
CCC2—coherence	4.12 ± 3.35 (3)	4.42 ± 3.14 (5)	0.342
CCC2—appropriateness of speech	5.20 ± 2.75 (5)	6.15 ± 2.72 (7)	0.064
CCC2—stereotyped language	5.55 ± 3.53 (5)	5.82 ± 3.33 (6)	0.366
CCC2—use of context	4.71 ± 3.27 (4)	6.24 ± 3.26 (6)	**0.021^a^**
CCC2—nonverbal communication	5.53 ± 2.70 (6)	6.12 ± 2.60 (6)	0.163
CCC2—social relations	4.24 ± 2.82 (4)	5.97 ± 2.54 (6)	**0.003^a^**
CCC2—interests	7.04 ± 3.38 (7)	7.15 ± 3.19 (7)	0.441**^a^**

^a^Independent samples *t*‐test, the bold values are considered statistically significant.

For all participants, TEDIL receptive language standard scores showed significant negative correlations with chronological age (*r* = –0.476) and television viewing time (*r* = –0.603). A moderate negative correlation was also found between AAT raw scores and chronological age (*r* = –0.333). Significant correlations were observed between gestational age and CCC‐2 syntax (*r* = 0.238); between birth weight and CCC‐2 speech (*r* = 0.313), semantics (*r* = 0.251), use of context (*r* = 0.301), nonverbal communication (*r* = 0.312), and social relationships (*r* = 0.234); and between maternal education and stereotyped language (*r* = 0.242) (Table 3).

**TABLE 3:** Factors associated with TEDIL, AAT, and CCC‐2 for all individuals.

	Gestational week at birth	Chronological age	Birth weight	Maternal gestational age	Maternal age	Maternal education	Television viewing duration
TEDIL receptive language standard scores	0.310^a^	−0.476**	0.248	0.041	0.012	0.093	−0.603**
TEDIL expressive language standard scores	0.064^a^	−0.286	0.012	0.260	0.237	−0.014	−0.285
AAT raw score	0.055	−0.333*	−0.163	−0.073	−0.108	−0.121	−0.002
AAT standard score	0.209	−0.180	0.180	−0.079	−0.069	0.161	−0.176
DILTAR	−0.209	0.166	−0.136	−0.163	−0.086	0.235	0.047
CCC2—speech	0.108	−0.089	0.313**	−0.057	−0.094	0.171	−0.004
CCC2—syntax	0.238*	0.030	0.172	−0.061	−0.077	0.098	0.144
CCC2—semantics	0.036	−0.093	0.251*	−0.235	−0.288*	0.153	−0.042
CCC2—coherence	0.003	0.043	0.057	−0.096	−0.140	0.050	0.025
CCC2—appropriateness of speech	0.105	−0.146	0.066	−0.163	−0.171	0.072	0.020
CCC2—stereotyped language	−0.015	0.046	0.055	−0.171	−0.190	0.242*	0.098
CCC2—use of context	0.098	−0.096	0.301**	0.106	0.056	0.061	−0.263
CCC2—nonverbal communication	0.079	0.108	0.312**	0.148	0.103	0.102	−0.088
CCC2—social relations	0.188	−0.050	0.234**	−0.156	−0.139	0.178	0.046
CCC2—interests	0.008	−0.130	0.128	−0.147	−0.182	0.176	0.090

^a^Pearson correlation test, the bold values are considered statistically significant.

*p* < 0.05*, *p* < 0.01** indicate significance.

Among twins, significant correlations were found between TEDIL receptive language standard scores and birth weight (*r* = 0.441), maternal gestational age (*r* = 0.484), maternal age (*r* = 0.487), and maternal education (*r* = –0.522) (*p* < 0.05). TEDIL expressive language standard scores were correlated with maternal education (*r* = –0.446, *p* < 0.05). CCC‑2 speech scores were significantly correlated with gestational week at birth (r = –0.332), maternal gestational age (r = –0.349), maternal age (r = –0.354), and maternal education (r = 0.488) (p < 0.05) (Table 4).

**TABLE 4:** Factors associated with TEDIL, AAT, and CCC‐2 for twins.

	Gestational week at birth	Chronological age	Birth weight	Maternal gestational age	Maternal age	Maternal education	Television viewing duration
TEDIL receptive language standard scores	0.152^a^	0.158	0.441*	0.484*	0.487*	−0.522*	−0.162
TEDIL expressive language standard scores	0.187^a^	0.073	−0.013	0.422	0.448	−0.446*	−0.408
AAT raw score	0.124	−0.183	0.179	−0.238	−0.208	−0.050	−0.556
AAT standard score	0.135	0.082	0.287	−0.300	−0.285	0.149	−0.298
DILTAR	−0.031	−0.099	−0.079	0.169	0.163	−0.066	0.117
CCC2—speech	−0.332*	0.042	0.000	−0.349*	−0.354*	0.488**	0.066
CCC2—syntax	0.065	0.017	0.379*	−0.395*	−0.362*	0.092	0.252
CCC2—semantics	0.300	0.023	0.341*	−0.187	−0.150	−0.226	0.000
CCC2—coherence	−0.004	0.101	0.320*	−0.473**	−0.448*	−0.053	0.229
CCC2—appropriateness of speech	0.102	0.094	0.151	−0.298	−0.290	−0.212	0.229
CCC2—stereotyped language	0.109	−0.090	0.227	−0.542**	−0.511**	−0.063	0.306
CCC2—use of context	−0.073	−0.042	0.193	−0.432*	−0.421*	0.258	0.153
CCC2—nonverbal communication	0.125	0.254	0.388*	−0.086	−0.054	−0.143	−0.089
CCC2—social relations	0.105	0.060	0.320*	−0.481**	−0.443*	0.093	−0.069
CCC2—interests	−0.061	−0.129	0.205	−0.372*	−0.366*	0.130	0.031

^a^Pearson correlation test, the bold values are considered statistically significant.

*p* < 0.05*, *p* < 0.01** indicate significance.

In twins, birth weight showed significant positive correlations with CCC‐2 subscales, including syntax, semantics, coherence, nonverbal communication, and social relationships. Significant negative correlations were found between maternal gestational age and CCC‐2 domains, as well as between maternal age and CCC‐2 domains such as speech, syntax, coherence, stereotyped language, social relationships, and interests (*p* < 0.05) (Table 4).

Among singletons, a moderate positive correlation was found between AAT raw scores and gestational age (*r* = 0.642). DILTAR results were correlated with birth weight (*r* = –0.343) and maternal education (*r* = 0.497). Additional significant correlations were observed between gestational age and CCC‐2 speech and appropriateness of speech (both showing negative correlations), maternal education and CCC‐2 speech (positive), and between CCC‐2 syntax and television viewing time (positive) (Table 5).

**TABLE 5:** Factors associated with TEDIL, AAT, and CCC‐2 for singletons.

	Gestational week at birth	Chronological age	Birth weight	Maternal gestational age	Maternal age	Maternal education	Television viewing duration
TEDIL receptive language standard scores	−0.156	−0.044	0.470	−0.072	−0.089	0.096	0.092
TEDIL expressive language standard scores	−0.104^a^	0.097	−0.103	0.821	0.900	−0.052	−0.018
AAT raw score	0.642*	−0.034	0.049	0.295	0.361	0.418	0.301
AAT standard score	−0.401	0.120	0.116	−0.402	−0.449	−0.700	−0.224
DILTAR	−0.201	0.238	−0.343*	0.072	0.061	0.497**	−0.101
CCC2—speech	−0.452*	0.010	−0.065	−0.052	−0.120	0.370*	−0.030
CCC2—syntax	−0.258	0.280	−0.073	−0.079	−0.103	0.151	0.374*
CCC2—semantics	−0.295	−0.009	−0.087	0.061	0.058	0.107	0.047
CCC2—coherence	−0.245	0.083	−0.104	0.336	0.318	0.164	0.098
CCC2—appropriateness of speech	−0.379*	−0.131	−0.079	0.021	−0.074	0.118	0.018
CCC2—stereotyped language	−0.146	0.071	0.054	−0.055	−0.124	0.258	−0.002
CCC2—use of context	−0.158	−0.050	0.133	−0.015	−0.000	0.286	−0.009
CCC2—nonverbal communication	0.100	0.104	0.071	0.219	0.175	0.214	0.051
CCC2—social relations	0.078	−0.119	0.026	0.122	0.099	−0.270	0.332
CCC2—interests	0.092	−0.317	0.077	0.106	0.030	−0.202	0.274

^a^Pearson correlation test, the bold values are considered statistically significant.

*p* < 0.05*, *p* < 0.01** indicate significance.

## 4 Discussion

This study not only examined the language development of twins but also evaluated their speech and communication skills, compared them with those of singletons, and investigated factors that could be related to all these skills in twins and singletons. Children aged 24–36 months, a critical period for development, were assessed and a comprehensive analysis was presented. Given both the scarcity of evidence on Turkish‐speaking twin populations and the relatively high frequency of twin births among multiple pregnancies reported in Türkiye (TÜİK 2024)—which makes this group both clinically and culturally significant—the present research provides culturally specific insights into variability in language, speech, and communication development. By addressing this gap, the study contributes population‐specific evidence that may inform early identification and intervention practices tailored to the Turkish context.

The study found significant differences between twins and singletons in child age, birth weight, and gestational age. These findings indicate that twins generally have lower birth weights and earlier gestational ages. The literature reports that twins are born approximately three weeks earlier than singletons and exhibit lower birth weights from 33 weeks onward (Bleker et al. 1979; Ullemar et al. 2015). Our results align with these findings, likely attributable to factors such as shared intrauterine environments, divided placental resources, and earlier maternal‐fetal physiological challenges in twin pregnancies (Giorgione et al. 2025; Santana et al. 2018).

Twins demonstrated significantly lower receptive language, articulation, and pragmatic communication performance compared to singletons. Specifically, twins showed weaker performance in the CCC‐2 subscales of speech, syntax, semantics, and social relationships, suggesting delays across multiple developmental domains. These results are consistent with prior studies highlighting an increased risk of language delays in twins (Rice et al. 2018). These differences may reflect the combined influence of biological and environmental factors. In the present study, twins had significantly lower gestational ages and birth weights compared to singletons. Prematurity and low birth weight are known to be associated not only with structural language delays but also with pragmatic communication difficulties and speech production challenges, likely due to immaturity in neural connectivity and early neurodevelopmental risk. Therefore, the weaker performance observed in speech, syntax, semantics, and social relationships may partly reflect prematurity‐related neurodevelopmental risk in addition to environmental influences. Environmental mechanisms may further contribute to this risk. Thorpe (2006) noted that reduced parent–child interaction and shared linguistic input in twins can negatively impact language development (Thorpe 2006). As an environmental factor, the necessity for parents to divide attention between two children may reduce the duration and quality of linguistic input for each child, potentially hindering language development (Rutter et al. 2003; Taylor et al. 2018; Thorpe 2006). When biological immaturity coexists with reduced one‐to‐one interaction, the cumulative effect may extend beyond language acquisition to broader communication and speech skills.

The study found that twins’ social relationships (CCC‐2) and speech skills (AAT) were significantly weaker than those of singletons, indicating that the twinness effect extends beyond language acquisition to encompass communication and speech skills in 2‐ to 3‐year‐olds. Laffey‐Ardley and Thorpe (2006) reported lower social and friendship scores among twins aged 3 to 6 years compared to singletons, a finding supported by our observation of lower social relationship scores in twins (Laffey‐Ardley and Thorpe 2006). Similarly, Hay et al. (1987) found weaker articulation skills in 30‐month‐old twin boys, which persisted even eight months after preschool enrollment (Hay et al. 1987). The similarity in age range between our study and that of Hay et al. strengthens the consistency of these findings. Rice et al. (2018) longitudinally examined the twinness effect in 4‐ to 6‐year‐olds and reported that it largely diminishes over time, with grammar outcomes increasingly influenced by hereditary factors (Rice et al. 2018). However, our findings suggest that during the early developmental window of 24–36 months—a period characterized by heightened neuroplasticity—the twinness effect may be more pronounced. This heightened risk in early toddlerhood may reflect the combined influence of biological immaturity and environmental constraints specific to twin rearing contexts. One potential explanation for these delays is cryptophasia, where twins develop a private, mutually understood but externally unclear language system (Bishop et al. 2003). While cryptophasia may represent a socially adaptive phenomenon within the twin dyad, it may also temporarily limit exposure to conventional linguistic models. Cultural and family interaction patterns may further shape how this phenomenon manifests and influences broader communication development.

The study's findings highlight the importance of several biological, sociodemographic, and environmental variables that were significantly associated with language, speech, and communication skills. When examining environmental variables, we observed that twins’ television viewing time was significantly longer than that of singletons and was negatively correlated with receptive language scores. These findings indicate a significant association between increased television viewing time and lower receptive language performance. The literature frequently highlights that passive screen time, such as television, reduces word exposure and face‐to‐face interaction opportunities, potentially impairing language development (Christakis 2009; Linebarger and Walker 2005; Rice et al. 2018). Consistent with this perspective, TEDIL receptive language standard scores were negatively correlated with both chronological age and television viewing time across all children. Additionally, a negative correlation between AAT raw scores and chronological age was observed. These patterns may suggest that, in the absence of sufficient environmental stimulation, language and speech development may not progress at the expected rate. Limiting screen time and providing appropriate supportive factors can make significant contributions to language and speech development (Reilly et al. 2007).

In the present study, with regard to sociodemographic variables, maternal education and maternal age were significantly associated with several language and communication measures. Maternal education was positively correlated with TEDIL receptive and expressive language scores in twins. Furthermore, positive correlations were observed between maternal education and CCC‐2 stereotyped language scores across all children, as well as DILTAR scores in singletons. Developmental psychology literature considers maternal education an indicator of access to cognitively stimulating experiences and enriched linguistic environments within the home (Entwislea and Astone 1994). In line with this perspective, the findings of the present study suggest that maternal education may represent an important sociodemographic variable associated with language outcomes. Higher maternal education levels may reflect greater capacity to provide developmentally appropriate linguistic stimulation, which may in turn be related to stronger language and communication skills in children. Maternal age was significantly associated with several language development measures, particularly in twins. Significant correlations were observed between maternal age and TEDIL receptive language raw scores, DILTAR results, and CCC‐2 subscales including speech, semantics, and appropriateness of speech. The literature suggests that maternal age influences perinatal outcomes like birth weight, indirectly affecting children's language development (Liu and Blair 2002; Zerbeto et al. 2015).

When evaluated in terms of biological variables, birth weight in twins was positively associated with TEDIL receptive language standard scores as well as with the syntax, semantics, and coherence subscales of the CCC‐2. In addition, CCC‐2 speech scores were significantly associated with gestational week at birth. In singletons, gestational age was associated with AAT raw scores, while birth weight was associated with DILTAR outcomes. These findings are consistent with the literature indicating that prematurity and low birth weight may be associated with increased developmental risk in language skills (Zerbeto et al. 2015). Taken together, these results suggest that biological maturity at birth appears to be associated with language and speech development in early childhood.

Taken together, the findings of the present study indicate that early language, speech, and communication development cannot be attributed to a single category of influences. The observed differences between twins and singletons, as well as the associations identified with environmental, sociodemographic, and biological variables, point to a multifactorial developmental process. In this context, genetic influences likely operate alongside environmental conditions rather than independently. Previous research has demonstrated that associations between family educational background and cognitive development reflect both environmental and genetic contributions (Rice et al. 2018; Trzaskowski et al. 2014). In twins, shared genetic backgrounds combined with overlapping environmental contexts further complicate the disentanglement of these influences. Therefore, the patterns observed in the present study are best interpreted within a dynamic gene–environment interaction framework. This perspective suggests that biological maturity at birth, parental characteristics, and environmental stimulation may function together within broader developmental systems rather than as isolated determinants. Future longitudinal and multivariate studies are needed to clarify the relative contribution and developmental trajectories of these interacting factors.

The study has some limitations. First, the effects of monozygotic versus dizygotic twinning on language development were not examined, overlooking potential zygosity effects. Gender differences in language development were alsonot explored. Additionally, the study groups were not homogeneous in birth weight, gestational age, and chronological age, increasing the likelihood that other biological and environmental factors, alongside the twinness effect, influence language development. In this respect, twinship and prematurity were inherently co‐occurring in our sample, making it difficult to isolate their independent contributions to the observed developmental differences. Finally, regression‐based predictive modeling was not performed, as the subgroup sizes did not meet the recommended subject‐to‐predictor ratio for stable multivariate analysis (Tabachnick et al. 2013) and substantial intercorrelations among key predictors (e.g., gestational age and birth weight) would have introduced multicollinearity. Future longitudinal studies with larger samples are warranted to examine the predictive contribution of biological, sociodemographic, and environmental factors to language development in twins and singletons.

A key strength of this study is its comprehensive evaluation of language, speech, and communication skills. The CATALISE report by Bishop et al. (2017) recommends that language assessments combine interviews with parents or caregivers, informal clinician observations, standardized tests, and criterion‐referenced evaluations (Bishop et al. 2017). Young children in this age group may not always cooperate fully with standardized tests, which can obscure their true language performance. To address this, this study utilized not only clinician‐administered tests but also parent‐reported assessments. Scales like DILTAR and CCC‐2, widely used in the literature, provide valuable data when children are uncooperative, offering a broader perspective on language development and enhancing the study's overall robustness through the use of multiple assessment tools.

In conclusion, this study provided culturally grounded evidence from Turkish children and demonstrated that 24‐ to 36‐month‐old twins were at higher risk of delays in language, speech, and communication skills compared to their singleton peers. The weaker articulation skills observed in twins indicated that the twin effect extended beyond language acquisition to speech skills as well. Therefore, comprehensive and multidimensional screening protocols are important for twins. Assessing twins' language, speech, and communication skills in early childhood may enable the timely detection of potential developmental delays. In addition, biological factors such as birth weight and genetic influences, as well as sociodemographic factors such as the maternal educational level and environmental factors such as television viewing time, were significantly associated with language, speech, and communication outcomes. This suggested that language and speech development in twins might need to be approached in a multifaceted manner, not solely based on biological factors. Early intervention approaches that combine speech‐language therapy with environmental support strategies might help reduce developmental delays and support communication skills.

## Author Contributions

**Mümüne Merve Parlak** contributed to conceptualization, investigation, methodology, validation, software, resources, visualization, Writing – original draft, and Writing – review & editing. **Emrah Karacaoğlu** contributed to conceptualization, data curation, project administration, methodology, and validation. **Cansu Yıldırım** contributed to writing – original draft, writing – review & editing, project administration, software, and methodology. **Oğuz Kadir Eğilmez** contributed to conceptualization, investigation, writing – review & editing, validation, project administration, and data curation. All authors read and approved the final version of the manuscript.

## Funding

The authors have nothing to report.

## Ethics Statement

All procedures performed in studies involving human participants were in accordance with the ethical standards of the institutional and/or national research committee and with the 1964 Helsinki declaration and its later amendments or comparable ethical standards. Before the recruitment, a written informed consent was obtained from each participant Ethical approval was granted by the Non‐Invasive Clinical Research Ethics Committee of Sakarya University Faculty of Medicine (Ethics Committee Date: 21.11.2024, No: 173).

## Conflicts of Interest

The authors have no conflict of interest to declare.

## Data Availability

All data generated or analyzed during this study are included in this article. Further enquiries can be directed to the corresponding author.
